# An empirical investigation of the potential impact of selective inclusion of results in systematic reviews of interventions: study protocol

**DOI:** 10.1186/2046-4053-2-21

**Published:** 2013-04-10

**Authors:** Matthew J Page, Joanne E McKenzie, Sally E Green, Andrew B Forbes

**Affiliations:** 1 School of Public Health and Preventive Medicine, Monash University, The Alfred Centre, 99 Commercial Road, Melbourne, VIC 3004, Australia

**Keywords:** Systematic review, Randomised controlled trials, Reporting, Bias, Research methodology

## Abstract

**Background:**

Systematic reviewers may encounter a multiplicity of outcome data in the reports of randomised controlled trials included in the review (for example, multiple measurement instruments measuring the same outcome, multiple time points, and final and change from baseline values). The primary objectives of this study are to investigate in a cohort of systematic reviews of randomised controlled trials of interventions for rheumatoid arthritis, osteoarthritis, depressive disorders and anxiety disorders: (i) how often there is multiplicity of outcome data in trial reports; (ii) the association between selection of trial outcome data included in a meta-analysis and the magnitude and statistical significance of the trial result, and; (iii) the impact of the selection of outcome data on meta-analytic results.

**Methods/Design:**

Forty systematic reviews (20 Cochrane, 20 non-Cochrane) of RCTs published from January 2010 to January 2012 and indexed in the Cochrane Database of Systematic Reviews (CDSR) or PubMed will be randomly sampled. The first meta-analysis of a continuous outcome within each review will be included. From each review protocol (where available) and published review we will extract information regarding which types of outcome data were eligible for inclusion in the meta-analysis (for example, measurement instruments, time points, analyses). From the trial reports we will extract all outcome data that are compatible with the meta-analysis outcome as it is defined in the review and with the outcome data eligibility criteria and hierarchies in the review protocol. The association between selection of trial outcome data included in a meta-analysis and the magnitude and statistical significance of the trial result will be investigated. We will also investigate the impact of the selected trial result on the magnitude of the resulting meta-analytic effect estimates.

**Discussion:**

The strengths of this empirical study are that our objectives and methods are pre-specified and transparent. The results may inform methods guidance for systematic review conduct and reporting, particularly for dealing with multiplicity of randomised controlled trial outcome data.

## Background

Systematic reviewers may encounter a multiplicity of outcome data in the reports of randomised controlled trials (RCTs) included in their reviews [1-3]. For example, within a single RCT report there may be data for the outcome depression based on multiple measurement scales (for example, the Hamilton rating scale for depression (HRSD) and the Beck depression inventory (BDI)), at multiple time points (for example, weeks three, six, and nine post intervention), and analysed in multiple ways (for example, as final and change from baseline values). When there is multiplicity of outcome data, the selection of data to include in the review should be based on a clinical or methodological rationale (or both), and ideally specified a priori. However, in some cases systematic reviewers may select results based on the magnitude, direction of effect, or statistical significance [1,3,4] (henceforth referred to as selective inclusion). Selective inclusion is problematic as it may misrepresent the available evidence, leading to selective inclusion bias [5,6].

An empirical study by Tendal *et al*. [3] suggested that multiplicity of outcome data in RCTs is common and the selected result to include may impact on the meta-analytic estimate. The authors investigated the extent of three sources of multiplicity - measurement instruments, time points, and intervention groups - in 83 RCTs included in 19 Cochrane reviews reporting a standardised mean difference (SMD) meta-analysis. In 18 (of 19) meta-analyses, at least one type of multiplicity was found in at least one included RCT. After extracting all RCT outcome data that were compatible with the inclusion criteria of the review protocol, Monte Carlo simulations were used to calculate all possible SMDs for each meta-analysis. The median difference between the smallest and largest meta-analytic SMD result was 0.40 (range 0.04 to 0.91), suggesting potential for large and important variability in meta-analytic results. The authors did not investigate whether there was an association between the included result and its characteristics (for example, statistical significance, magnitude), or the impact of other types of multiplicity (for example, multiple analyses such as intention-to-treat and per-protocol) [3].

Concerns about the potential for selective inclusion have led to initiatives to minimise its occurrence. The Cochrane Collaboration Methodological Expectations for Cochrane Intervention Reviews (MECIR) initiative and the Institute of Medicine Committee on Standards for Systematic Reviews of Comparative Effectiveness Research have recently published guidance recommending that systematic reviewers report detailed protocols that pre-specify eligible outcome measurement instruments and time points for inclusion in the review [7,8]. An optional field to provide information on eligible measurement instruments and time points is also available on the registration form of PROSPERO, an international online prospective register of systematic reviews launched in February 2011 [9,10]. Tendal *et al*. also recommend that systematic reviewers pre-specify a hierarchy of measurement instruments and time points when multiplicity of outcome data is anticipated (for example, pre-specifying that HRSD data will be included in a meta-analysis of depression if both HRSD and BDI data are reported in studies) [3]. Tendal *et al*. suggested that systematic reviewers have not consistently reported such detailed protocols. For example, while all of the 19 Cochrane protocols reported eligible measurement instruments, none reported a hierarchy of measurement instruments, eight (42%) reported eligible time points and only one (5%) reported a hierarchy of time points [3]. These protocols were published prior to 2006 and no studies have since assessed the frequency of pre-specification of these and other types of outcome data eligibility criteria and hierarchies (for example, preferring adjusted rather than unadjusted effect estimates). Furthermore, no studies have assessed whether systematic review protocols affect selective inclusion of results in systematic reviews.

Another initiative that may minimise potential selective inclusion of results is the development of standardised sets of outcomes (known as core outcome sets) to collect in clinical trials of a specific condition [11]. Establishing a core outcome set in RCTs can inform which outcomes should be included in systematic reviews [12,13]. The earliest core outcome sets were developed in the 1990s, for rheumatoid arthritis (RA) and osteoarthritis (OA) in the 1990s [14-18]. Through the work of the Core Outcome Measures in Effectiveness Trials (COMET) initiative [19], core outcome sets are currently being developed for a range of other conditions. In addition to the core outcomes in RA and OA studies, recommended measurement instruments are also available (for example, the Health Assessment Questionnaire to measure function in RA RCTs [20], and a hierarchy of pain measurement instruments for use in OA systematic reviews, where a global pain score is preferred over a pain on walking score if data for both instruments are available in an RCT report) [21-23]. In contrast, similar guidance does not exist for other conditions that have neither agreed core outcome sets nor core measurement instruments (for example, depressive and anxiety disorders) [24-26]. To date there has been no evaluation of whether core outcome sets affect selective inclusion of results in systematic reviews.

To our knowledge, no prior work has quantitatively assessed the evidence for potential bias in meta-analytic results, which can occur when reviewers selectively include results from the set available. Quantifying this potential for bias is important as the results of meta-analyses are used by various stakeholders to inform clinical practice and policy decisions. The aim of this study is to investigate, in a cohort of systematic reviews, the potential impact of selective inclusion of RCT results on meta-analytic effects. The primary objectives of this study are to investigate: 1) how often there is multiplicity of outcome data in RCT reports (for example, arising from multiple measurement scales, time points, and analyses); 2) the association between the RCT outcome data included in the meta-analysis and the magnitude and statistical significance of the RCT result, and 3) the impact of the selection of RCT outcome data on meta-analytic results.

The secondary objectives are to: 1) quantify how many systematic review protocols report outcome data eligibility and hierarchies, and 2) explore how potential selective inclusion of results is modified by (i) the existence of a systematic review protocol, and (ii) a core outcome set being available for the clinical condition under review.

## Methods/design

### Overview of the study

Forty systematic reviews (20 Cochrane, 20 non-Cochrane) of RCTs published from January 2010 to January 2012 and indexed in the Cochrane Database of Systematic Reviews (CDSR) or PubMed will be randomly sampled. The first meta-analysis of a continuous outcome within each review will be included. From each review protocol (where available) and published review we will extract information regarding which types of outcome data were eligible for inclusion in the meta-analysis (for example, measurement instruments, time points, analyses). From the RCT reports we will extract all outcome data that are compatible with the meta-analysis outcome as it is defined in the review and with the outcome data eligibility criteria and hierarchies in the review protocol. The association between selection of RCT outcome data included in a meta-analysis and the magnitude and statistical significance of the RCT result will be investigated. We will also investigate the impact of the selected trial result on the magnitude of the resulting meta-analytic effect estimates.

### Eligibility criteria

A systematic review was defined using the definition by Moher *et al*.: …‘the authors’ stated objective was to summarize evidence from multiple studies, and the article described explicit methods, regardless of the details provided’ [27]. The eligibility criteria for inclusion of both Cochrane and non-Cochrane systematic reviews include: 1) the review was published between Issue 1, 2010 to Issue 1, 2012 in the CDSR, or between January 2010 to January 2012 in a non-Cochrane journal; 2) the review is written in English (as we do not have the resources available to translate systematic reviews published in other languages); 3) references of all included RCTs are reported in the review; 4) the review evaluates the effects of any intervention for either RA, OA, depressive disorders (including major depressive disorder, dysthymic disorder, bipolar depression, seasonal affective disorder, and post-partum depression), or anxiety disorders (including generalized anxiety disorder, obsessive-compulsive disorder, panic disorder, phobic disorders, acute stress disorder, and post-traumatic stress disorder) [28], and 5) the review includes at least one continuous outcome meta-analysis of RCTs (for example, pain, function, number of tender or swollen joints, depression, anxiety, quality of life), with reporting of i) either the summary statistics (for example, mean, SD) or effect estimate and precision of each RCT included in the meta-analysis, and ii) the meta-analytic effect estimate and its precision.

We have selected these clinical areas to explore whether the existence of a core outcome set being available for the clinical condition of the review (namely, RA and OA) impacts on selective inclusion of results. We will focus on continuous outcomes since there is greater scope for multiplicity of continuous outcomes in these clinical areas (for example, arising from multiple measurement instruments, final versus change from baseline values, adjusted versus unadjusted means, sub-scale scores) compared with dichotomous outcomes. Both Cochrane and non-Cochrane reviews will be eligible regardless of whether a published protocol for the review is available. Unpublished protocols will be requested from authors. Both new and updated reviews will be eligible. For updated reviews, the protocol drafted closest to the latest update will be included in this study.

The exclusion criteria are: 1) no meta-analyses of continuous outcomes are reported in the review; 2) results from non-randomised studies are included in each of the meta-analyses of continuous outcomes, and 3) non-standard meta-analytical methods are used (for example, Bayesian, multiple-treatments, or individual patient data meta-analyses).

### Literature search

We will identify systematic reviews by performing an electronic search of the CDSR and PubMed. We will use RA and OA search terms recommended by The Cochrane Collaboration Musculoskeletal Review Group [29], and depressive and anxiety disorders search terms recommended by The Cochrane Collaboration Depression, Anxiety and Neurosis Review Group [30]. For the PubMed search strategy we will combine the clinical search terms with a search filter used to identify systematic reviews in a previous empirical study on the epidemiology and reporting characteristics of systematic reviews [27]. As the CDSR only includes records of Cochrane reviews, we will not use the systematic review search filter in the CDSR search strategy. We will limit searches to English language publications and date of publication from 1 January 2010 to 31 January 2012. The search strategies for both databases are reported in Additional file 1.

### Selection of systematic reviews

The citations retrieved from the CDSR and PubMed databases will be exported to Microsoft Excel and randomly sorted using the random number generator (citations of Cochrane reviews retrieved in the PubMed search will be deleted). One investigator (MJP) will read down the list of randomly sorted citations and screen the titles and abstracts, marking them as potentially eligible or ineligible. The full text of each potentially eligible systematic review will be retrieved and assessed against the inclusion criteria. This process will continue until 10 Cochrane RA or OA reviews, 10 non-Cochrane RA or OA reviews, 10 Cochrane depressive or anxiety disorders reviews, and 10 non-Cochrane depressive or anxiety disorders reviews, are included. Within both clinical categories (that is, RA or OA and depressive or anxiety disorders), we will not constrain the selection by the particular clinical condition (for example, we will not require an equal number of reviews of depression and anxiety). Any difficulties with determining whether a systematic review meets inclusion criteria will be resolved by discussion with a second researcher (JEM).

### Selection of continuous outcome for investigation

We will select from each systematic review the first meta-analysis of a continuous outcome that meets the inclusion criteria (henceforth referred to as the index meta-analysis). The index meta-analysis may be selected from the abstract, summary of findings table, or results section of the review, depending on where the result is first reported in the publication. We will not constrain the selection based on the outcome label of the review (that is, primary, secondary, or unlabelled), because we anticipate that in some reviews the primary outcome(s) may be dichotomous or the primary continuous outcome may not have been meta-analysed. We will not constrain the selection based on the domain measured (for example, pain, or function). Meta-analyses will be eligible regardless of meta-analytic effect measure (that is, MD or SMD), meta-analytical model (that is, fixed-effect or random-effects), and number of RCTs included (as long as at least two RCTs are included).

### Report retrieval

We will retrieve reports of systematic reviews, review protocols, and RCTs using library services. Reports of RCTs may comprise journal articles, conference abstracts, unpublished dissertations, or regulatory agency or pharmaceutical company reports. For RCTs included in Cochrane reviews with reports written in languages other than English, we will request a copy of the translation, if available, from the Cochrane Review Groups, or will use Google Translate. We will retrieve reports of RCTs included in the index meta-analysis and those reported by the systematic reviewers as investigating the same pairwise comparison but which were excluded from the meta-analyses (to explore whether any eligible outcome data may have been missed from these reports or potentially excluded based on the results). If more than one reference for an RCT was reported by the systematic reviewers (for example, both a journal article and a conference abstract), we will retrieve all references reported. This will enable investigation of potential selective inclusion resulting from differences in results reported across different sources [31-33].

### Data extraction

One investigator (MJP) will extract data from all reviews and RCTs into a standardised form created in Microsoft Excel. This form will be pilot-tested on one review from each of the four categories (Cochrane RA or OA review, non-Cochrane RA or OA review, Cochrane depression or anxiety disorders review, non-Cochrane depression or anxiety disorders review), and refined accordingly. A second investigator will independently extract data from a random sample of 10 reviews and their included RCTs. If many data extraction discrepancies are identified, we will consider undertaking double data extraction for the remaining reviews. Any discrepancies between the data extracted will be resolved through discussion or adjudication by a third investigator if necessary. The list of data we will extract from the systematic review protocols, published systematic reviews, and RCTs is reported in Additional file 2. A brief summary is provided below.

#### Data to extract from systematic review protocols

From the systematic review protocol (where available) we will extract: 1) general characteristics of the review, including date of publication, and participants, interventions, comparisons, and outcomes of interest to the review; 2) reported outcome data eligibility criteria (for example, measurement scales, time points, intervention groups, and/or analyses), and 3) reported outcome data hierarchies (for example, whether final values were preferred over change from baseline values if both are reported in an RCT publication).

#### Data to extract from published systematic reviews

From the published systematic review, we will extract the same information as from the protocols. In addition, we will extract information on any other outcome data reported in the review that are related measures of the index meta-analysis outcome under the same comparison. For example, if the index meta-analysis outcome is global pain at 4 to 6 weeks, we will record whether any outcome data for different pain scales at different time points were included in the review, either in a subsequent meta-analysis or in separate tables; these additional analyses also include sensitivity analyses related to the index meta-analysis. For the index meta-analysis, we will extract the following information: 1) the measurement instrument, time point of measurement, and intervention and comparison group for each RCT; 2) summary statistics for both groups in each RCT; 3) the MD or SMD, measures of variability, the statistical significance, and direction of the effect estimate for each RCT and for the meta-analytic effect; 4) heterogeneity statistics, and 5) which outcome data were obtained from the trialists because it was not reported in the RCT publication, involved algebraic manipulation of statistics (for example, calculating SDs from reported 95% CIs of the mean), came from a report translated into English, or required a method of imputation (such as imputing a missing SD).

#### Data to extract from RCT reports

From the RCT reports we will extract all outcome data that are compatible with the index meta-analysis outcome as it is defined in the review and with the outcome data eligibility criteria and hierarchies reported in the review protocol. This could include data from multiple measurement instruments measuring the same outcome, multiple time points, multiple intervention or control groups, final and change from baseline values, intention-to-treat and per-protocol analyses, adjusted and unadjusted effect estimates, and other analyses. For example, if the index meta-analysis is an MD meta-analysis of depression scores and the systematic reviewers report in the protocol that only HRSD outcome data will be included in a meta-analysis of depression, and specify no other outcome data eligibility criteria, we will extract all data for the HRSD (for example, all time points, adjusted and unadjusted effect estimates), but no data for any other depression measurement instrument reported in the RCTs. Alternatively, if the index meta-analysis is an SMD meta-analysis of pain intensity at 12 weeks, and the systematic reviewers have not pre-specified any outcome data eligibility criteria or hierarchies, we will extract all pain intensity data (for example, based on any measurement scale, intention-to-treat and per-protocol analyses) from each RCT at 12 weeks only. For systematic reviews without a protocol, we will request the unpublished protocol from the systematic reviewers. If one does not exist or is not provided, we will assume that no outcome data eligibility criteria or hierarchies were pre-specified, and will extract all outcome data from the RCTs, as long as they are compatible with the index meta-analysis outcome as it is defined in the review (as per the second example above). Final and change from baseline values are a special case in that systematic reviewers performing an SMD meta-analysis of different measurement instruments should include only final values or change from baseline values, not a mixture [34]. For systematic reviews that only include final values in an SMD meta-analysis, we will not extract any change from baseline values from the RCTs (and vice versa for systematic reviews that only include change from baseline values in an SMD meta-analysis). If systematic reviewers include a mixture of final and change from baseline values in an SMD meta-analysis, we will extract both types of values from the RCTs.

For each type of RCT outcome data deemed eligible for inclusion in the meta-analysis, we will extract: 1) the measurement instrument, time point of measurement, and intervention and comparison groups; 2) sample sizes, measures of central tendency, and measures of variability per group; 3) the effect estimate (MD or SMD) and measures of variability, the statistical significance, and direction of the effect estimate; 4) the baseline SD of the outcome per group, and 5) whether outcome data were fully reported in the RCT report (where fully reported is defined as reporting sufficient information to include the data in a meta-analysis [35]). We will use DigitizeIt 1.5.8© software to extract outcome data presented in figure format when the data are not available in the text of the report. We will not contact trialists for unpublished data.

### Sample size

A study of the characteristics of meta-analyses (with at least two studies) contained in the January 2008 issue of the *Cochrane Database of Systematic Reviews*[36] found the median number of studies per meta-analysis to be three. Assuming three RCTs per meta-analysis, a sample of forty meta-analyses will provide one hundred and twenty RCTs. This will allow estimation of the proportion of RCTs with multiplicity of outcome data to within ± 9% of the true population percentage. This assumes a population proportion of 50%, a worst case scenario for the sample size calculation.

### Analysis

#### Descriptive analyses of general characteristics of systematic reviews

We will use descriptive statistics to summarise the characteristics of the systematic reviews included in the study. These characteristics include, for example, the clinical condition, intervention and comparison type, number of primary and secondary outcomes (reported in the review protocol and published review), number of RCTs included in the review overall, and characteristics of the index meta-analysis outcome (outcome definition, meta-analytic effect measure, meta-analytical model, and number of included RCTs).

#### Descriptive analyses of reporting of outcome data eligibility criteria and hierarchies in systematic review protocols and published reviews

We will calculate the proportion of systematic review protocols and published reviews reporting at least one outcome data eligibility criterion and the proportion reporting at least one outcome data hierarchy. We will also separately calculate the proportion of protocols and reviews reporting eligibility criteria and hierarchies in relation to each of the following types of outcome data multiplicity: 1) multiple measurement instruments; 2) multiple time points; 3) multiple intervention or control groups; 4) final and change from baseline values; 5) sets of participants contributing to the analysis (for example, intention-to-treat, per-protocol, as-treated); 6) unadjusted and adjusted effect estimates; 7) period results in crossover RCTs, and 8) other. Further, we will calculate the proportion of systematic reviews with at least one discrepancy in outcome data eligibility criteria and hierarchies between the protocol and published review (where a discrepancy is defined as an addition, removal, or modification of an eligibility criterion or hierarchy).

#### Quantifying outcome data multiplicity in RCT reports

We will calculate the proportion of RCTs with at least one type of outcome data multiplicity that is compatible with the index meta-analysis outcome as it is defined in the review and with the outcome data eligibility criteria and hierarchies reported in the review protocol. We will also calculate the proportion of RCTs with the following types of outcome data multiplicity: 1) multiple measurement instruments; 2) multiple time points; 3) multiple intervention or control groups; 4) final and change from baseline values; 5) sets of participants contributing to the analysis (for example, intention-to-treat, per-protocol, as-treated); 6) unadjusted and adjusted effect estimates; 7) period results in crossover RCTs, and 8) other. In addition, for each RCT we will quantify the number of effect estimates that were eligible for inclusion in the index meta-analysis, and will quantify the median (interquartile range) of eligible effect estimates per RCT. We will also quantify the number of eligible effect estimates that were not included in the index meta-analysis but were included in other meta-analyses or elsewhere in the review (for example, tables).

#### Testing the association between selection of outcome data and the magnitude and statistical significance of the effect estimate

When multiple effect estimates are available for inclusion in a meta-analysis, without pre-specified selection rules, several different methods may be acceptable (in terms of not introducing bias) for selecting an effect estimate from the set available. These mechanisms may include: 1) selecting data for the most commonly reported instrument, time point, or analysis across RCTs; 2) random selection of an effect estimate; 3) selection of the median effect estimate, and 4) selection of the outcome data based on clinical criteria. The commonality of these selection methods is that the selection of the effect estimate is not based on choosing systematically higher or lower effect estimates. If across the RCTs, selection methods 1) to 4) are employed, we would expect that the distribution of selected effect estimates would be consistent with what we would observe under purely random selection, although this does not necessarily mean that the process used to select the effect estimates was indeed random selection.

We have developed an index, which we call the Potential Bias Index (PBI), to assess whether the estimates selected for inclusion in the index meta-analysis are systematically higher or lower than what would be expected by purely random selection. This index is based on the ordered effect estimates for each trial and the positioning (that is, rank) of the effect estimate selected within that order. A rank of 1 is assigned to the smallest effect estimate and a rank equal to the number of effect estimates is assigned to the largest effect estimate. Since the number of effect estimates varies across trials we rescale the ranks of the effect estimates to reflect their relative positioning (in ranking units) between the smallest and largest effect estimates. This is obtained by subtracting one from the rank of the selected effect estimate and dividing by the number of effect estimates minus one. The smallest effect estimate in a trial then has a location of zero and the largest effect estimate has a location of 1. So for a trial with three effect estimates and the rank of the chosen effect estimate of 2, its location is (2–1)/(3–1) = 0.5 - halfway between the lowest and highest rank. The Potential Bias Index (PBI) is defined as the weighted average of the locations of the selected estimates for each trial, with the weights being the number of effect estimates in each trial. With this weighting, greater priority is given to the locations of effect estimates the larger the number of effect estimates there were to choose from. The expression for PBI is:

$$\mathrm{PBI}=\sum_{i=1}^{k}\frac{n_{i}\left(X_{i}-1\right)}{n_{i}-1}/\sum_{i=1}^{k}n_{i}$$

where there are *k* trials, *n*_*i*_ is the number of effect estimates in trial *i*, and *X*_*i*_ is the rank of the selected effect estimate in trial *i*. Derivation of this index and a worked example is provided in Additional files 3 and 4. Only trials with more than one effect estimate are included in the PBI since a trial with one effect estimate provides no information about relative location. When the largest effect estimate in each of the trials is selected for inclusion, the PBI will have the value 1, and conversely PBI = 0 when the smallest effect estimate is always selected. Under a process consistent with random selection, the PBI is expected to take the value of 0.5, so, on average the chosen effect estimates are at the middle location. Similarly, a PBI of 0.75 would indicate that on average the effect estimates chosen were 75% of the distance between the smallest and largest ranks, or equivalently halfway between the middle and highest rank. We have constructed a simple statistical test based on the PBI to test whether the observed selection of effect estimates is consistent with randomness of selection (see Additional file 3). Confidence intervals for the PBI can be constructed using bootstrap methods by resampling individual trials [37]. We will also apply the PBI to assess possible selection mechanisms in which the smaller *P*-values of the effect estimates are chosen for inclusion.

#### Impact of selection of outcome data on meta-analytic results

The PBI described above will also be used to compare the index meta-analytic effect estimates with all possible meta-analytic effects. For each meta-analysis, all possible meta-analytic effects will be calculated from all combinations of available RCT effect estimates. The meta-analysis model used to combine the estimates (either fixed or random effects) will be the model that was used in the systematic review. However, sensitivity analyses will be undertaken to examine whether the type of meta-analysis model affects the PBI.

We will also investigate the impact of the selected RCT effects on the magnitude of the resulting meta-analytic effect estimates. For each meta-analysis, the difference between the index meta-analytic effect estimate and the median of all possible meta-analytic effect estimates will be calculated. These differences will be standardised (by dividing by the pooled baseline SD of the outcome) and meta-analysed using a random-effects model across reviews. The meta-analytic weights will be based on the standardised standard error of the median meta-analytic estimates, and between RCT variability estimated using DerSimonian and Laird’s method of moments estimator [38]. Note that this approach ignores the correlation between the meta-analytic effects within meta-analysis, arising from correlated RCT effects.

#### Subgroup analyses

We will examine whether the existence of 1) a systematic review protocol and 2) a core outcome set being available for the clinical condition of the review affects a) the specificity of outcome data eligibility criteria and hierarchies reported in systematic review protocols and published reviews; b) the proportion of RCTs with multiplicity and the proportion of systematic reviews with at least one RCT with multiplicity; c) the PBI of the RCT effect estimates selected for inclusion in the index meta-analysis, and d) the PBI of the resulting index meta-analytic effect estimates.

#### Sensitivity analyses

For systematic reviews without protocols, it is not known whether the outcome eligibility criteria reported in the methods section of the review were specified prior, or subsequent to undertaking the review. Therefore, for our primary analyses, we have chosen to include the set of RCT effect estimates that are compatible with the assumption of no pre-specified outcome data eligibility criteria. However, through sensitivity analyses, we plan to investigate if the PBIs (calculated at both the RCT and meta-analysis level) are modified when the set of RCT effect estimates are restricted to those that are compatible with the outcome data eligibility criteria and hierarchies specified in the methods section of the review.

## Discussion

To our knowledge, this is the first empirical study designed to investigate the association between selection of RCT outcome data included in a meta-analysis and the magnitude and statistical significance of the RCT result. In publishing this protocol we are following the lead of others who have encouraged the pre-specification and transparent reporting of the objectives and design of methodological studies [39-43].

There are several strengths of our study. We will use systematic review methods to identify eligible reviews, including use of explicit inclusion criteria, sensitive search strategies, duplicate selection of reviews, and standardised and pilot-tested data extraction forms. We will perform double data-extraction on a random sample of reviews and their included RCTs, and will consider performing this on the complete sample if the data extraction discrepancy rate is high. In addition to exploring whether there is evidence of selective inclusion of RCT results in systematic reviews, we will examine what the potential impact of this is on meta-analytic estimates.

There are also several limitations to our study. We are focusing only on meta-analyses of continuous outcomes, and so will not investigate potential selective inclusion arising from types of multiplicity unique to dichotomous outcomes (for example, binary events defined in multiple ways, or continuous measurement instruments dichotomised using different cut-points). Our study is also limited to systematic reviews of RA, OA, depressive disorders and anxiety disorders. Some of the continuous outcomes likely to be included in our study (for example, pain, function, and quality of life) exist in systematic reviews of other conditions (such as low-back pain), but our findings may have limited generalisability to other clinical areas. However, our focus on continuous outcomes and these clinical areas enables us to examine the impact of core outcome sets on selective inclusion of results. Finally, our study will only investigate the existence of potential bias in meta-analytic effect estimates that can result from systematic reviewers’ selective inclusion of results reported by trialists. It is possible that the effect estimate(s) available in an RCT publication may have been selectively reported by the trialists (for example, data collected using other measurement scales may have been omitted based on the results). Therefore, both selective reporting by trialists and selective inclusion by systematic reviewers may in combination bias the results of a meta-analysis [6]; however, our analysis will only examine the latter.

Meta-analysis results are of interest to various stakeholders and are used to inform clinical practice and policy decisions. If the results of meta-analyses are biased by selective inclusion of results, additional methods guidance for systematic review conduct and reporting will be necessary. Systematic review organisations have only recently recommended that systematic reviewers pre-specify eligible measurement instruments and time points in their protocols [7,8]. This advice may need to be extended to encompass other common types of outcome data multiplicity.

## Abbreviations

BDI: Beck depression inventory; CDSR: Cochrane Database of Systematic reviews; HRSD: Hamilton rating scale for depression; IOM: Institute of Medicine; MD: Mean difference; MECIR: Methodological Expectations for Cochrane Intervention Reviews; OA: Osteoarthritis; PBI: Potential Bias Index; RA: Rheumatoid arthritis; RCT: Randomised controlled trial; SMD: Standardised mean difference.

## Competing interests

MJP has roles within The Cochrane Collaboration including systematic review trainer for the Australasian Cochrane Centre, member of the Bias Methods Group, Statistical Methods Group, and Trainer’s Network, and author of Cochrane systematic reviews. JEM has roles within The Cochrane Collaboration including Co-convenor of the Statistical Methods Group, member of the Methods Board and the Bias Methods Group, Statistical Editor of the Consumers and Communication Review Group, and author of Cochrane systematic reviews. SEG has roles within The Cochrane Collaboration including Co-Director of the Australasian Cochrane Centre, past editor of the Cochrane Handbook of Systematic Reviews of Interventions, and author of Cochrane systematic reviews managed by the Cochrane Collaboration Musculoskeletal Review Group (though not rheumatoid arthritis or osteoarthritis reviews). ABF has no competing interests to declare.

## Authors’ contributions

MJP and JEM conceived the study design. MJP wrote the first draft, with contributions from JEM. SEG and ABF provided input on the study design. ABF developed the Potential Bias Index, test statistics, and simulations. All authors contributed to revisions of the manuscript and take public responsibility for its content.

## Supplementary Material

Additional file 1 Search strategies. Click here for file

Additional file 2 Data to be collected from systematic review protocols, published systematic reviews, and randomised controlled trial (RCT) reports. Click here for file

Additional file 3 Mathematical details of the construction of the Potential Bias Index (PBI) and associated statistical test. Click here for file

Additional file 4 Worked example of the Potential Bias Index. Click here for file
