# Supplementary material for: An empirical investigation of the potential impact of selective inclusion of results in systematic reviews of interventions: study protocol
Source: Syst Rev. 2013 Apr 10;2:21. doi: 10.1186/2046-4053-2-21 (PMC3626625; doi:10.1186/2046-4053-2-21)
Supplement: Additional file 1 — Search strategies. [file 2046-4053-2-21-S1.doc]

**Additional file 1: Search strategies**

*Cochrane Database of Systematic Reviews (Wiley Interscience (Online) interface) search strategy*

1. degenerative arthritis[tw]
2. “Arthritis, Rheumatoid”[MeSH]
3. rheumatoid arthritis[tw]
4. rheumatism[tw]
5. “Arthritis, Juvenile Rheumatoid”[MeSH]
6. caplan's syndrome[tw]
7. felty's syndrome[tw]
8. rheumatoid[tw]
9. ankylosing spondylitis[tw]
10. arthrosis[tw]
11. sjogren*[tw]
12. “Osteoarthritis”[MeSH]
13. Osteoarthr*[tw]
14. #1 or #2 or #3 or #4 or #5 or #6 or #7 or #8 or #9 or #10 or #11 or #13 or #14
15. “Depression”[MeSH]
16. “Anxiety”[MeSH]
17. “Anxiety Disorders”[MeSH]
18. depress*[tw]
19. dysthymi*[tw]
20. anxiety[tw] OR anxious[tw]
21. #15 or #16 or #17 or #18 or #19 or #20
22. #14 or #21
23. #22 Limits: English, Publication Date from 2010/01/01 to 2012/01/31

*MEDLINE (PubMed interface) search strategy*

1. degenerative arthritis[tw]
2. “Arthritis, Rheumatoid”[MeSH]
3. rheumatoid arthritis[tw]
4. rheumatism[tw]
5. “Arthritis, Juvenile Rheumatoid”[MeSH]
6. caplan's syndrome[tw]
7. felty's syndrome[tw]
8. rheumatoid[tw]
9. ankylosing spondylitis[tw]
10. arthrosis[tw]
11. sjogren*[tw]
12. “Osteoarthritis”[MeSH]
13. osteoarthr*[tw]
14. #1 or #2 or #3 or #4 or #5 or #6 or #7 or #8 or #9 or #10 or #11 or #13 or #14
15. “Depression”[MeSH]
16. “Anxiety”[MeSH]
17. “Anxiety Disorders”[MeSH]
18. depress*[tw]
19. dysthymi*[tw]
20. anxiety[tw] OR anxious[tw]
21. #15 or #16 or #17 or #18 or #19 or #20
22. #14 or #21
23. cochrane database syst rev[ta] or search[tw] or meta-analysis[pt] or medline[tw] or systematic review[tw]
24. (meta-analysis[pt] or meta-analysis[tw] or meta-analysis[mesh] or review[pt] or search*[tw]) and methods[ab]
25. #23 or #24
26. #22 and #25
27. #26 Limits: English, Publication Date from 2010/01/01 to 2012/01/31
