# Supplementary material for: An empirical investigation of the potential impact of selective inclusion of results in systematic reviews of interventions: study protocol
Source: Syst Rev. 2013 Apr 10;2:21. doi: 10.1186/2046-4053-2-21 (PMC3626625; doi:10.1186/2046-4053-2-21)
Supplement: Additional file 2 — Data to be collected from systematic review protocols, published systematic reviews, and randomised controlled trial (RCT) reports. [file 2046-4053-2-21-S2.doc]

**Additional file 2: Data to be collected from systematic review protocols, published systematic reviews, and randomised controlled trial (RCT) reports**

From each systematic review protocol we will extract:

- the type of systematic review (Cochrane or non-Cochrane);
- the date of publication;
- the clinical condition investigated;
- the intervention(s) investigated;
- the comparison(s) investigated
- the list of primary outcomes reported in the method section;
- the list of secondary outcomes reported in the methods section;
- the list of outcomes not labelled as primary or secondary in the methods section;
- text justifying the exclusion of specific outcomes from the review (e.g. reporting that surrogate outcomes reported in eligible RCTs will not be included in the review because they were of limited clinical relevance);
- eligibility criteria for outcome data (e.g. reporting which measurement scales, time points, intervention groups, and analyses are eligible for inclusion in the systematic review);
- text regarding any hierarchies of outcome data preferred for inclusion in the review (e.g. reporting that intention-to-treat analyses are preferred over per-protocol analyses if both are reported in a RCT publication);
- any reference to the use of core outcomes when selecting outcomes and outcome data to include in the review.

From each published systematic review, we will extract:

- the type of systematic review (Cochrane or non-Cochrane);
- the date of publication;
- the Cochrane Review Group (if a Cochrane review);
- whether the review is a new or updated review;
- the clinical condition investigated;
- the intervention(s) investigated;
- the comparison(s) investigated;
- the total number of RCTs included in the systematic review;
- the list of primary outcomes reported in the methods section;
- the list of secondary outcomes reported in the methods section;
- the list of outcomes not described as primary or secondary in the methods section;
- text justifying the exclusion of specific outcomes from the review;
- eligibility criteria for outcome data (e.g. reporting which measurement scales, time points, intervention groups, and analyses are eligible for inclusion in the systematic review);
- text regarding any hierarchies of outcome data preferred for inclusion in the review (e.g. reporting that intention-to-treat analyses are preferred over per-protocol analyses if both are reported in a RCT publication);
- any reference to the use of core outcomes when selecting outcomes to include in the review;
- text justifying the modification of outcomes from the protocol to publication (e.g. adding, omitting, upgrading, or downgrading an outcome description as primary or secondary);
- the title and definition of the continuous outcome meta-analysis selected for investigation (index meta-analysis);
- titles and definitions of on any other outcome data reported in the review that are alternative measures of the index meta-analysis outcome under the same comparison;
- total number of other outcomes reported in the review that are alternative measures of the index meta-analysis outcome;
- whether the index meta-analysis is a primary, secondary, or unlabeled outcome;
- the number of RCTs included in the index meta-analysis;
- text regarding the outcome measurement instrument, time point of measurement, and intervention and comparison group for each study effect;
- all reported summary statistics for each group in each study (mean, standard deviation, sample size, standard error of the mean, 95%CI of the mean);
- whether the included study outcome data is quantified as final or change from baseline values;
- whether the included study outcome data is analysed based on an intention-to-treat or an alternative analysis;
- the type of individual study and meta-analytic effect estimate reported (mean difference (MD) or standardized mean difference (SMD));
- the individual study and meta-analytic effect estimate results and measures of variability (standard error of the mean difference, 95%CI of the MD);
- the exact P-value or an indication of the statistical significance of the difference between groups on the outcome if the 95% confidence interval or exact P-value are not reported, for each individual study and for the meta-analytic effect (note that the P-value is assumed to be two-tailed; if stated that it is one-tailed, this will be noted and the reported P-value will be multiplied by two);
- the direction of the individual study and meta-analytic effect estimates (rated favours intervention, favours comparison, or no difference);
- the tau-squared value;
- the chi-squared value;
- the P-value for the chi-squared value;
- the I-squared statistic;
- how the meta-analysis has been reported in the review (e.g. forest plot, table, narrative description in text);
- text regarding which (if any) outcome data was included in the meta-analysis after having obtained this data from the trialists (because it was not reported in the RCT publication), or after transforming the data (e.g. calculating standard deviations from reported 95%CIs of the mean), or after translation of the non-English-language publication into English, or by using a method of imputation
- text regarding funding of the systematic review;
- text regarding any declarations of interest of the systematic reviewers.

From each RCT publication, we will extract for all eligible outcome data included in or excluded from the meta-analysis:

- text regarding the outcome measurement instrument, time point of measurement, and intervention and comparison groups on which the outcome data is based;
- sample sizes per group on which the outcome data is based;
- measures of central tendency for each group (mean, median);
- measures of variability for each group (standard deviation, standard error of the mean, 95%CI of the mean, interquartile range, range);
- whether the outcome data is quantified as final or change from baseline values;
- whether the outcome data is analysed based on an intention-to-treat or an alternative analysis;
- the type of effect estimate (MD or SMD);
- the effect estimate result and measures of variability (standard error of the MD, 95%CI of the MD);
- the exact P-value or an indication of the statistical significance of the difference between groups on the outcome if the 95% confidence interval or exact P-value are not reported (note that the P-value is assumed to be two-tailed; if stated that it is one-tailed, this will be noted and the reported P-value will be multiplied by two);
- the direction of the effect estimate (rated as “favours intervention”, “favours comparison”, or “no difference”);
- the t-test or Z-test value;
- the baseline mean and standard deviation of the outcome for each eligible group;
- whether the effect estimate was unadjusted or adjusted (and if adjusted, which variables were adjusted for);
- the location of the outcome data in the RCT publication;
- whether outcome data was fully reported in the RCT publication, and if not, which specific outcome data is required to be able to include that data in the meta-analysis.
