# Supplementary material for: An empirical investigation of the potential impact of selective inclusion of results in systematic reviews of interventions: study protocol
Source: Syst Rev. 2013 Apr 10;2:21. doi: 10.1186/2046-4053-2-21 (PMC3626625; doi:10.1186/2046-4053-2-21)
Supplement: Additional file 3 — Mathematical details of the construction of the Potential Bias Index (PBI) and associated statistical test. [file 2046-4053-2-21-S3.doc]

**Additional file 3: Mathematical details of the construction of the Potential Bias Index (PBI) and associated statistical test**

Here we provide the mathematical details of a summary statistic which we call the Potential Bias Index (PBI). In brief, PBI measures the average location of the rank of the selected effect estimates on a scale from 0 to 1, where 0 represents the lowest possible rank and 1 represents highest possible rank, and 0.5 represents the middle value. Consider a situation with *k* trials, labeled i=1,2, …, k, and for the *i’th* trial there are *n_i_* effect estimates. Order these effect estimates from smallest to largest, with the smallest getting rank 1 and the largest rank n_i_ . Let X_i_ denote the rank of the effect estimate that was actually chosen for reporting in trial i. Rescale X_i_ so that it can take values between 0 and 1, with the value 0 when X_i_ is the lowest rank and the value 1 when X_i_ is the highest rank. Call this variable Y_i_ and define it as
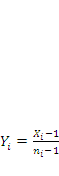
. We define the PBI as a weighted average of the Y_i_’s , with weights equal to the number of effect measures in each trial n*i*,


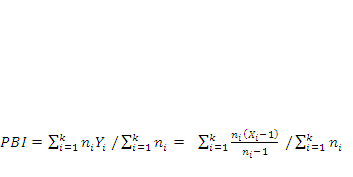

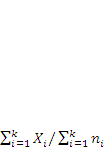


Under an assumption of random selection, X_i_  could be any one of the effect estimates with equal probability and therefore
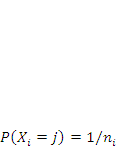
 for each j=1,2,…,n_i_. It is then straightforward to calculate that the expected value of X_i_ under randomness is
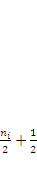
, the expected value of Y_i_ is 0.5, and therefore E[PBI] = 0.5. Similarly, the variance of PBI can be calculated as

$Var(PBI)=\frac{\sum_{i=1}^{k} \frac{n_{i}^{2}(n_{i}+1)}{12(n_{i}-1)}}{\left(\sum_{i=1}^{k} n_{i}\right)^{2}}$

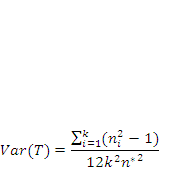

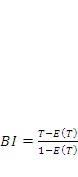
 A statistical test of the null hypothesis of randomness can be constructed using
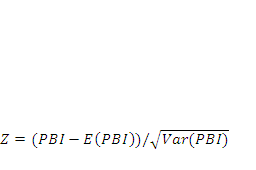

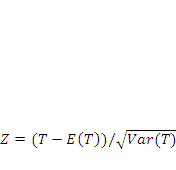
, which should follow a standard normal distribution under the hypothesis of randomness when the number of trials is sufficiently large.

We assessed the properties of this statistical test under the assumption of randomness by simulating a set of trials, each with a number of effect estimates, and then randomly selecting one of the effect estimates with equal probability and comparing its ranking with the other effect sizes. Specifically, we generated between k=3 and k=120 trials, with the number of effect estimates per trial chosen from a truncated Poisson distribution of mean 3 or 6, where the truncation excluded values of zero or one effect estimate so that each trial contained two or more effect estimates. The reported effect estimate was then randomly chosen from the set of effect estimates for each trial. The statistic PBI was then calculated for each set of trials, and assessment of its statistical significance at the 5% level made by conversion to the test statistic Z as above and comparison with the standard normal distribution. Over 20,000 replications of each configuration, the percentage of Z statistics where the hypothesis of randomness was rejected was

recorded, i.e. the Type I error rate. The simulation standard error for a nominal 5% Type I error rate with 20,000 replications was +/- 0.15%:

| Number of trials | Average number of effect measures per trial | Empirical Type I error rate % |
| --- | --- | --- |
| 3 | 3 | 4.86 |
| 3 | 6 | 4.61 |
| 5 | 3 | 4.58 |
| 5 | 6 | 4.83 |
| 10 | 3 | 5.08 |
| 10 | 6 | 5.11 |
| 15 | 3 | 4.83 |
| 15 | 6 | 5.01 |
| 30 | 3 | 5.03 |
| 30 | 6 | 4.92 |
| 60 | 3 | 5.04 |
| 60 | 6 | 4.92 |
| 120 | 3 | 4.81 |
| 120 | 6 | 5.14 |

The results indicate that the nominal Type I error of 5% is well preserved using this test even for assessments with as few as 3 trials.

*Technical Note: Our definition of*
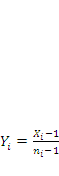
 *in some disciplines (e.g. education, social science, and spread sheet software) is occasionally called the “percentile ranking”, however percentiles have a slightly different definition in the statistical and health sciences and therefore we have refrained from providing any interpretation of Y or PBI in terms of such “percentile ranks”.*
