# Supplementary material for: An empirical investigation of the potential impact of selective inclusion of results in systematic reviews of interventions: study protocol
Source: Syst Rev. 2013 Apr 10;2:21. doi: 10.1186/2046-4053-2-21 (PMC3626625; doi:10.1186/2046-4053-2-21)
Supplement: Additional file 4 — Worked example of the Potential Bias Index. [file 2046-4053-2-21-S4.doc]

Additional file 4: Worked example of the Potential Bias Index.

Consider a review consisting of 10 trials. The number of effect estimates for each trial is provided as well as the rank of the effect estimate chosen among those available for each trial. Suppose the data are the following:

| Trial | Number of effect estimates (n) | Rank of chosen effect estimate (X) | Location of chosen effect estimate (Y) |
| --- | --- | --- | --- |
| 1 | 3 | 2 | 0.50 |
| 2 | 7 | 6 | 0.83 |
| 3 | 4 | 3 | 0.67 |
| 4 | 6 | 5 | 0.80 |
| 5 | 4 | 2 | 0.33 |
| 6 | 5 | 4 | 0.75 |
| 7 | 6 | 6 | 1 |
| 8 | 2 | 1 | 0 |
| 9 | 4 | 4 | 1 |
| 10 | 5 | 4 | 0.75 |

For Trial #1, there were 3 effect estimates and the rank of the chosen effect estimate was 2, that is the middle or median value, and its location is therefore halfway between the lowest and highest rank. For Trial #2 there were 7 effect estimates and the chosen estimate had rank 6. There are a total of 6 units of rank between 1 and 7 (i.e. 1 to 2, 2 to 3, 3 to 4, 4 to 5, 5 to 6 and 6 to 7) and the chosen rank of 6 is therefore 5/6ths = 83% of the distance between lowest and highest rank. In general, the rank location Y is calculated as (X-1)/(n-1).

The statistic PBI = $PBI=\frac{3\times0.50+7\times0.83+\cdots+4\times1+5\times0.75}{3+7+4+\cdots+4+5}=\frac{33.61}{46}=0.73$

Therefore on average the effect estimates chosen were 73% of the distance between the smallest and largest rank, i.e. approximately halfway between the middle rank and the maximum.

The standard error of the PBI can be calculated to be 0.118, and therefore the Z-statistic equals (0.73-0.50)/0.118 = 1.97, with a two-tailed p-value of 0.049. This indicates some evidence that the effect estimate selection is systematically higher than that expected by random selection.

A 95% confidence interval for the PBI is obtained from 1000 bootstrap replications as 0.58 to 0.85.
